# Catheter Ablation vs. Medical Therapy for Ventricular Tachycardia in Ischemic Cardiomyopathy: A Meta-Analysis and Trial Sequential Analysis of Randomized Controlled Trials

**DOI:** 10.31083/RCM46164

**Published:** 2026-02-26

**Authors:** Su-Ping Wang, Yuan Yuan, Peng-Yu Zhong, Zhen-Yu Zhou

**Affiliations:** ^1^Department of Cardiology, The Second Clinical Medical College of North Sichuan Medical College, Beijing Anzhen Nanchong Hospital of Capital Medical University & Nanchong Central Hospital, 637000 Nanchong, Sichuan, China

**Keywords:** catheter ablation, ventricular tachycardia, ischemic heart disease, implantable cardioverter defibrillator, meta-analysis

## Abstract

**Background:**

Managing ischemic cardiomyopathy-related ventricular tachycardia (VT) remains clinically challenging since no definitive consensus exists regarding the optimal therapeutic approach. Therefore, this study aimed to assess the safety and efficacy of catheter ablation for VT in patients with ischemic cardiomyopathy.

**Methods:**

We systematically searched the PubMed, EMBASE, and Cochrane Library databases to identify pertinent clinical trials. We selected the relative risk (RR) and mean difference (MD) as the effect measures, which were calculated using Review Manager software. Additionally, we used trial sequential analysis to assess each outcome.

**Results:**

Our study included six randomized controlled trials with 1064 patients. Catheter ablation was found to reduce the risk of the composite endpoint (RR 0.83, 95% confidence interval (CI) 0.74–0.94; *p* = 0.002), cardiac hospitalizations (RR 0.82, 95% CI 0.71–0.95; *p* = 0.007), and adverse events (RR 0.75, 95% CI 0.62–0.91; *p* = 0.003). Additionally, no significant differences were observed between the two groups regarding VT recurrence (RR 0.94, 95% CI 0.83–1.06; *p* = 0.33), appropriate implantable cardioverter-defibrillator (ICD) shocks (RR 0.85, 95% CI 0.72–1.01; *p* = 0.06), or all-cause mortality (RR 0.93, 95% CI 0.73–1.18; *p* = 0.53).

**Conclusions:**

Catheter ablation reduced the incidence of composite endpoints, cardiac hospitalizations, and adverse events related to VT in patients with ischemic cardiomyopathy. However, no statistically significant differences were found between the two groups for VT recurrence, appropriate ICD shocks, and all-cause mortality.

**The PROSPERO Registration:**

https://www.crd.york.ac.uk/PROSPERO/view/CRD420251011744.

## 1. Introduction

Ischemic cardiomyopathy (ICM), characterized by left ventricular dysfunction resulting from severe coronary artery disease, is marked by high morbidity and all-cause mortality [1, 2]. As a common complication in patients with ICM, ventricular tachycardia (VT) significantly increases the incidence of sudden cardiac death [3]. The mechanisms underlying VT in ICM involve slow conduction zones formed in myocardial necrosis and reentrant circuits within fibrotic scar tissues [4, 5]. This electrophysiological substrate poses distinct therapeutic challenges. The 2017 AHA/ACC/HRS guidelines for VT management in ICM assign a class I recommendation to antiarrhythmic drugs (AADs), whereas catheter ablation receives a class IIb recommendation (Level C) as first-line therapy [6]. However, although AADs can reduce the frequency of VT episodes, their adverse effects are notable [7]. In particular, amiodarone may induce thyroid dysfunction and pulmonary toxicity, often leading to treatment discontinuation [8].

With ongoing exploration into VT management in ICM, catheter ablation has become an attractive intervention strategy. By eliminating VT foci and disrupting reentrant circuits, catheter ablation can effectively terminate VT episodes and ameliorate electrophysiological disturbances [9, 10]. Sapp *et al*. [11] performed a multicenter randomized controlled trial to explore whether catheter ablation is more effective than AADs in patients with VT. The results showed that the initial ablation strategy significantly decreased the risk of the primary composite endpoints, which included all-cause mortality, VT storms, and appropriate implantable cardioverter-defibrillator (ICD) shocks (HR (hazard ratio) 0.75, *p* = 0.03). Similarly, these clinical benefits were found in other randomized controlled trials [12, 13, 14]. However, catheter ablation may lead to various acute complications, and the procedure-related mortality cannot be overlooked [14, 15]. There remains ongoing debate regarding whether catheter ablation should be considered the primary therapeutic option from the outset.

Therefore, our study evaluated the efficacy and safety of catheter ablation versus medical therapy as initial interventions for VT in ICM patients. Additionally, we applied trial sequential analysis (TSA) to increase the credibility of our conclusions.

## 2. Materials and Methods

This meta-analysis followed the Preferred Reporting Items for Systematic Reviews and Meta-Analyses (PRISMA) guidelines [16] and was registered with PROSPERO (CRD420251011744, https://www.crd.york.ac.uk/PROSPERO/view/CRD420251011744). Ethical approval was not required.

### 2.1 Data Sources, Inclusion and Exclusion Criteria

To identify all relevant trials, we searched PubMed, EMBASE, and the Cochrane Library. The PubMed search strategy utilized the following terms: (“myocardial ischemia” OR “ischemic heart disease”) AND (“tachycardia, ventricular” OR “ventricular tachycardia”) AND (“catheter ablation” OR “ablation, catheter”) AND “randomized controlled trial”. The search dates ranged from inception to March 15, 2025. No language restrictions were applied. The complete search strategies for all databases are provided in **Supplementary Tables 1,2,3**.

The inclusion criteria were defined as follows: (a) Population: patients with ICM and VT; (b) Intervention: ICD implantation with catheter ablation; (c) Comparison: ICD implantation alone or ICD with AADs; (d) Outcomes: reporting of one or more predefined outcomes, including VT recurrence, the composite endpoint, appropriate ICD shocks, adverse events, all-cause mortality, and cardiac hospitalizations. The exclusion criteria were as follows: (a) Non-randomized controlled trials; (b) Duplicate publications; (c) Studies with insufficient data.

### 2.2 Data Extraction

We used NoteExpress software (Version 3.0, Beijing Aegean Software Company, Beijing, China) to filter out duplicate literature. The two reviewers (SPW and YY) independently assessed titles and abstracts to exclude ineligible studies, with any disagreements settled by consensus or third-author adjudication (ZYZ). Finally, the two reviewers independently extracted the relevant data, including: (a) article characteristics: publication year, country, and patients’ inclusion criteria; (b) population characteristics: mean age, sex, and the classification of cardiac function; (c) follow-up time and reported outcomes.

### 2.3 Evaluation of Study Quality and Outcomes

Bias assessment was conducted using the Cochrane Risk of Bias Tool, which evaluates seven specific domains: random sequence generation, allocation concealment, blinding of participants and personnel, incomplete outcome data, blinding of outcome assessors, selective outcome reporting, and other biases. Additionally, we employed the Grades of Recommendations Assessment, Development and Evaluation (GRADE) framework to gauge the quality of the evidence for individual outcomes [17, 18].

The primary outcome was defined as the recurrence of ventricular tachycardia (VT recurrence). Secondary outcomes included: (1) the composite endpoint, which is mainly composed of VT storms, appropriate ICD shocks, and mortality. There were different definitions of composite endpoints among the included studies, which are shown in the **Supplementary Table 4**; (2) appropriate ICD shocks; (3) all-cause mortality; (4) cardiac hospitalizations; (5) adverse events.

### 2.4 Data Synthesis 

We conducted the statistical analysis using Review Manager version 5.4 (Revman, The Cochrane Collaboration, Oxford, UK). For dichotomous outcomes, the relative risk (RR) and the mean difference (MD) were selected as the effect measures. The effect size was presented with a 95% confidence interval (95% CI). The *p*-value of the Chi-square test was used to assess heterogeneity, and I^2^ to describe the degree of heterogeneity. When *p* < 0.1, significant heterogeneity was found. The I^2^ values of 25%, 50%, and 75% represent low, moderate, and high heterogeneity, respectively [19]. The fixed-effects model was employed for meta-analysis when *p* ≥ 0.1. Conversely, when *p* < 0.1, subgroup analyses were conducted to explore sources of heterogeneity. If the heterogeneity sources could not be identified, we employed the random-effects model for the meta-analysis. We assessed publication bias using visual funnel plots. Additionally, the Trial Sequential Analysis (version 0.9.5.10; Copenhagen Trial Unit, Copenhagen, Denmark) was used to evaluate the results.

## 3. Results

### 3.1 Search Results and Baseline Characteristics

**Fig. 1.**
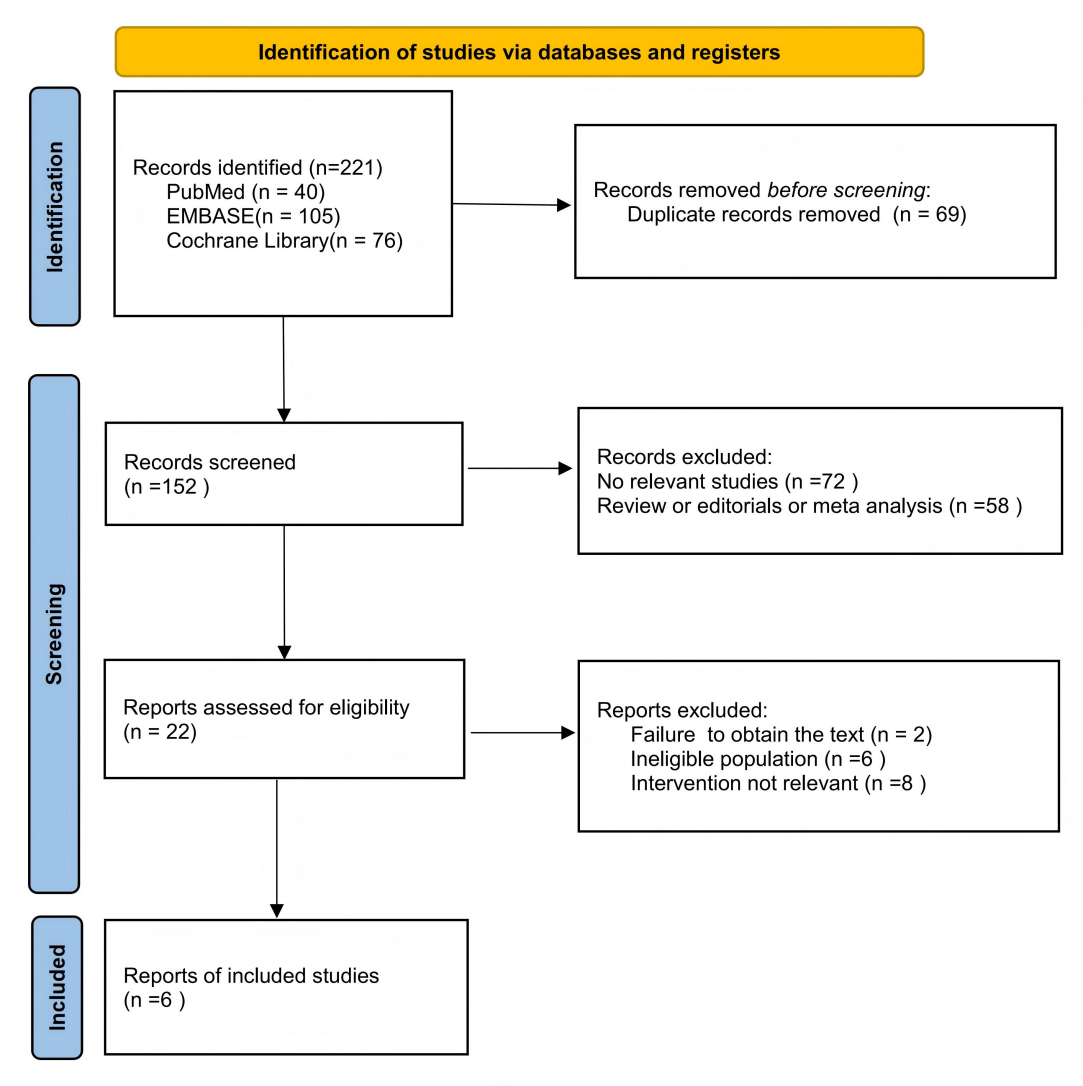
**The retrieval flowchart**.

We retrieved 221 articles from PubMed, EMBASE, and the Cochrane Library databases. After careful review, six randomized controlled trials met the eligibility criteria [11, 12, 13, 14, 20, 21]. The retrieval flowchart is shown in Fig. 1. Baseline characteristics were balanced between the ablation and control groups, as detailed in Table 1 (Ref. [11, 12, 13, 14, 20, 21]). The key features of the included trials are summarized in Table 2 (Ref. [11, 12, 13, 14, 20, 21]). The follow-up time was between 6 and 66 months. The mean left ventricular ejection fraction (LVEF) reported in the studies varied from 23% to 35%.

**Table 1.** **Baseline characteristics of the patients included**.

Study	Age		Male (%)		LVEF (%)		NYHA class I/II/III (%)		Time since last myocardial infarction (years)		Prior PCI		Prior CABG		ICD type (single-chamber/Dual-chamber ICD/CRT)	
	Ablation group	Control group	Ablation group	Control group	Ablation group	Control group	Ablation group	Control group	Ablation group	Control group	Ablation group	Control group	Ablation group	Control group	Ablation group	Control group
VTACH 2010 [13]	67.7	64.4	96.0	91.0	34.0	34.1	-	-	12.6	13.3	26	24	26	22	34/-/-	37/-/-
CALYPSO 2015 [20]	64.0	65.0	100.0	86.0	25.0	23.0	22/33/11	21/36/21	-	-	6	8	8	8	1/8/4	6/6/2
VANISH 2016 [14]	67.0	70.3	93.2	92.9	31.1	31.2	33/69/30	28/68/31	15.7	15.7	50	62	63	55	43/60/29	44/61/22
SMS 2017 [21]	68.4	65.9	87.0	81.0	32.0	30.4	-	-	-	-	46	46	41	43	-	-
SURVIVE-VT 2022 [12]	70.0	71.0	98.6	93.2	35.0	33.0	31/33/6	31/37/5	14.0	14.0	18	12	26	26	55/5/11	54/5/13
VANISH-2 2025 [11]	67.7	68.4	95.1	92.5	34.0	34.3	89/99/17	89/107/17	13.3	14.8	128	121	82	88	66/94/43	77/100/36

Abbreviations: CABG, coronary artery bypass grafting; ICD, implantable cardioverter-defibrillator; CRT, cardiac resynchronization therapy; PCI, percutaneous coronary intervention; LVEF, left ventricular ejection fraction; NYHA, New York Heart Association.

**Table 2.** **Baseline characteristics of the included trials**.

Study	Country	Design	Sample size	Patient inclusion criteria	Ablation group	Control group	Follow-up time (month)	Outcomes
VTACH 2010 [13]	Czechia, Denmark, Germany, Switzerland	RCT	107	Coronary artery disease (including prior MI and LVEF ≤50%), and documented stable clinical VT.	catheter ablation + ICD implantation	ICD implantation	24	VT recurrence, All-cause mortality, Appropriate ICD shocks adverse events, Cardiac hospitalizations
CALYPSO 2015 [20]	United States	RCT	27	Ischemic heart disease (with wall motion abnormality ≥70% coronary stenosis), and ≥1 ICD shock or ≥3 ATP therapies for monomorphic VT within 6 months.	catheter ablation + ICD implantation	AAD therapy + ICD implantation	6	VT recurrence, adverse events, Cardiac hospitalizations, All-cause mortality
VANISH 2016 [14]	Canada, Europe, United States, Australia	RCT	259	Previous myocardial infarction, and a VT episode within the past 6 months during treatment with amiodarone or other class I/III AADs.	catheter ablation + ICD implantation	escalated AAD therapy + ICD implantation	24	Composite primary endpoint, Cardiac hospitalizations, Appropriate ICD shocks, adverse events, All-cause mortality
SMS 2017 [21]	Denmark, Germany	RCT	111	Coronary artery disease with LVEF ≤40%, and clinically unstable spontaneous VT.	catheter ablation + ICD implantation	ICD implantation	33	VT recurrence, adverse events, Cardiac hospitalizations, Appropriate ICD shocks, All-cause mortality
SURVIVE-VT 2022 [12]	Spain	RCT	144	Previous myocardial infarction, and had an episode of very symptomatic VT.	catheter ablation +ICD implantation	AAD therapy + ICD implantation	24	VT recurrence, adverse events, Composite primary endpoint, Appropriate ICD shocks, Cardiac hospitalizations, All-cause mortality
VANISH-2 2025 [11]	Canada, United States, France	RCT	414	Previous myocardial infarction and had at least one of the VT events within the preceding 6 months while not being treated with AADs.	catheter ablation + ICD implantation	AAD therapy +ICD implantation	66	VT recurrence, adverse events, Composite primary endpoint, Appropriate ICD shocks, Cardiac hospitalizations, All-cause mortality

Abbreviations: ICD, implantable cardioverter-defibrillator; MI, myocardial infarction; RCT, randomized controlled trial; VT, ventricular tachycardia; LVEF, left ventricular ejection fraction; AADs, antiarrhythmic drugs; ATP, anti-tachycardia pacing.

### 3.2 The Primary Outcomes

**Fig. 2.**
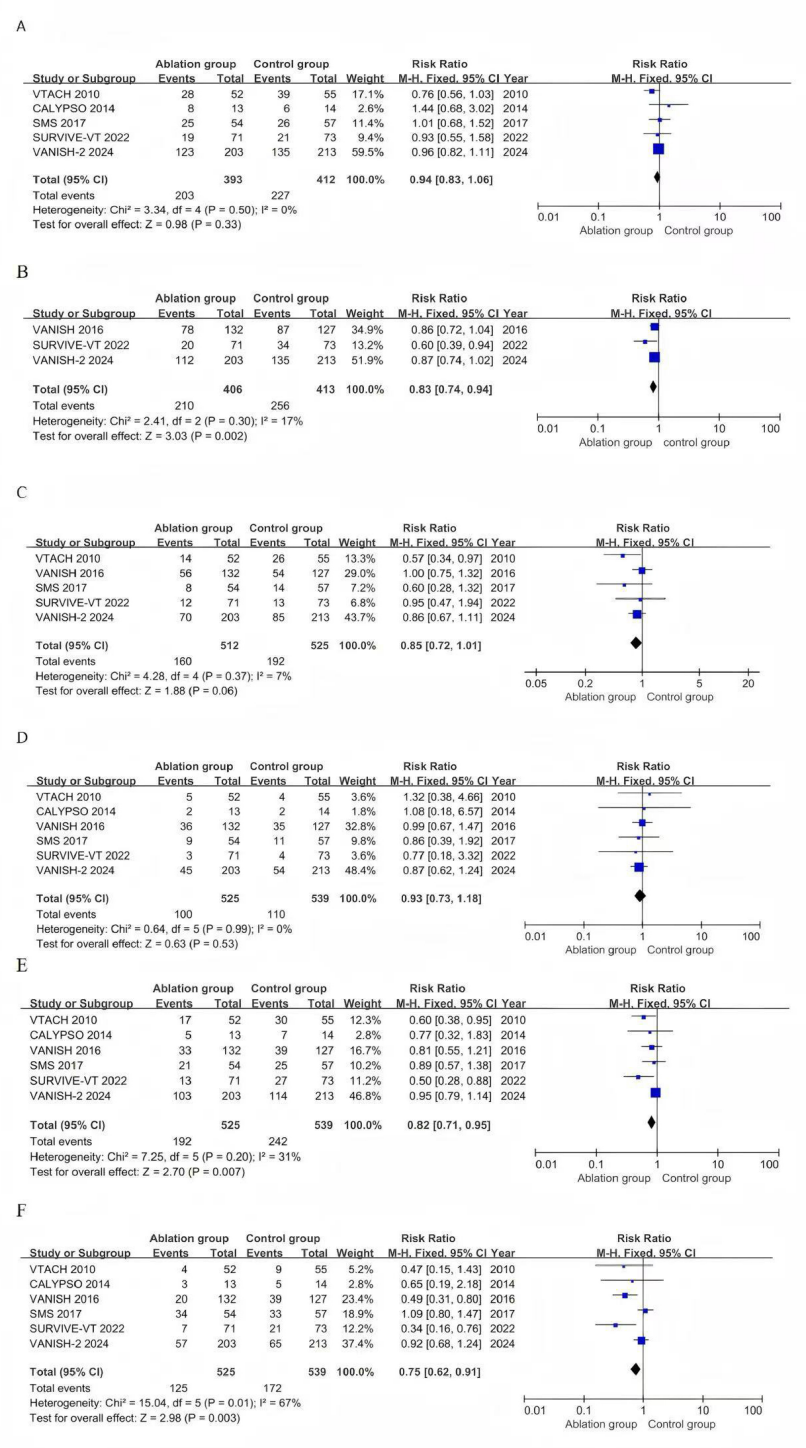
**Pooled analyses of catheter versus medical therapy for the outcomes**. (A) Forest plot of VT recurrence. (B) Forest plot of composite endpoints. (C) Forest plot of appropriate ICD shocks. (D) Forest plot of all-cause mortality. (E) Forest plot of cardiac hospitalizations. (F) Forest plot of adverse events. CI, confidence interval.

A total of 5 studies reported VT recurrence with 805 patients (Fig. 2A). The ablation group had a lower incidence of VT recurrence compared with the control group (RR 0.94, 95% CI 0.83–1.06, *p* = 0.33, I^2^ = 0%, *p *_h⁢e⁢t⁢e⁢r⁢o⁢g⁢e⁢n⁢e⁢i⁢t⁢y_ = 0.50).

### 3.3 Secondary Outcomes

The composite endpoint, reported in 3 studies involving 819 patients (Fig. 2B), showed a significantly lower incidence in the ablation group than in the control group (RR 0.83, 95% CI 0.74–0.94, *p* = 0.002; I^2^ = 17%, *p *_h⁢e⁢t⁢e⁢r⁢o⁢g⁢e⁢n⁢e⁢i⁢t⁢y_ = 0.30). Five studies involving 1037 patients reported the incidence of appropriate ICD shocks (Fig. 2C). No significant difference was observed between the two groups (RR 0.85, 95% CI 0.72–1.01, *p* = 0.06, I^2^ = 7%, *p *_h⁢e⁢t⁢e⁢r⁢o⁢g⁢e⁢n⁢e⁢i⁢t⁢y_ = 0.37).

The risk of all-cause mortality was reported across all trials (Fig. 2D), and no significant differences or heterogeneity were observed between the ablation and control groups (RR 0.93, 95% CI 0.73–1.18, *p* = 0.53, I^2^ = 0%, *p *_h⁢e⁢t⁢e⁢r⁢o⁢g⁢e⁢n⁢e⁢i⁢t⁢y_ = 0.99). A total of six studies reported the incidence of cardiac hospitalizations, including 1064 patients (Fig. 2E). In contrast to the control group, the ablation group had a lower risk of cardiac hospitalizations (RR 0.82, 95% CI 0.71–0.95, *p* = 0.007, I^2^ = 31%, *p *_h⁢e⁢t⁢e⁢r⁢o⁢g⁢e⁢n⁢e⁢i⁢t⁢y_ = 0.20).

Six studies with 1064 patients reported the incidence of adverse events (Fig. 2F). Fewer adverse events were observed in the ablation group than in the control group (RR 0.75, 95% CI 0.62–0.91, *p* = 0.003). Statistical heterogeneity was found in adverse events (I^2^ = 67%, *p*_h⁢e⁢t⁢e⁢r⁢o⁢g⁢e⁢n⁢e⁢i⁢t⁢y_ = 0.01). We conducted subgroup analyses according to follow-up time, LVEF, and pharmacologic interventions. In the ≤ 2-year follow-up subgroup, the ablation group had a lower risk of adverse events (RR 0.46, 95% CI 0.32–0.66, *p* < 0.0001, I^2^ = 0%, *p *_h⁢e⁢t⁢e⁢r⁢o⁢g⁢e⁢n⁢e⁢i⁢t⁢y_ = 0.82). However, the incidence of adverse events did not decrease in the > 2-year follow-up subgroup (RR 0.98, 95% CI 0.78–1.22, *p* = 0.83, I^2^ = 0%, *p *_h⁢e⁢t⁢e⁢r⁢o⁢g⁢e⁢n⁢e⁢i⁢t⁢y_ = 0.42). Furthermore, there was a significant difference between the two subgroups (*p *_i⁢n⁢t⁢e⁢r⁢a⁢c⁢t⁢i⁢o⁢n_ = 0.0005). No statistical difference was observed in the other subgroups (**Supplementary Table 5**). Additionally, we performed sensitivity analyses by excluding the SMS and VANISH-2 trials (RR 0.46, 95% CI 0.32–0.66, *p* < 0.0001, I^2^ = 0%, *p *_h⁢e⁢t⁢e⁢r⁢o⁢g⁢e⁢n⁢e⁢i⁢t⁢y_ = 0.82). Furthermore, we also conducted statistical analyses on other related outcomes (**Supplementary Table 6**).

### 3.4 Assessment of Quality, Trial Sequential Analysis, and Publication Bias

Each trial’s quality assessment and GRADE evidence are provided in the supplementary materials (**Supplementary Fig. 1** and **Supplementary Table 7**). The GRADE evidence quality assessments for the composite endpoint and cardiac hospitalizations are high, while those for other outcomes are moderate. The results of the TSA are presented in the **Supplementary Fig. 2**. The cumulative Z-curves for the composite endpoint (required information size: 3147), cardiac hospitalizations, and adverse events crossed the conventional significance boundary but neither crossed the TSA monitoring boundary nor reached the required information size. In contrast, the outcomes for VT recurrence, all-cause mortality, and appropriate ICD shocks did not cross either the conventional or the TSA monitoring boundaries. Furthermore, the assessment of publication bias using funnel plots revealed symmetric distributions of data points across all evaluated outcomes, indicating a low risk of bias (**Supplementary Fig. 3**).

## 4. Discussion

This meta-analysis included six randomized controlled trials and 1064 patients. The results showed that ablation reduced the incidence of the composite endpoint, cardiac hospitalizations, and adverse events related to VT in patients with ICM. However, no statistical differences were found in VT recurrence, all-cause mortality, and appropriate ICD shocks between the two groups.

VT in ICM is a life-threatening arrhythmia with a poor prognosis, with untreated patients facing a two-year mortality rate of 30% [22, 23]. Even after conventional treatment, patients still confront VT recurrence and ICD shocks [24]. Thus, the management of VT in ICM remains a clinical challenge. As the cornerstone of secondary prevention for sudden cardiac death, the ICD can terminate life-threatening arrhythmias, but it is unable to reduce or prevent VT episodes [25, 26]. AADs are accompanied by serious adverse reactions and drug resistance issues, which make their long-term efficacy controversial [27]. Catheter ablation can directly eliminate the foci with abnormal impulse formation or the reentrant circuits, thereby improving myocardial structural abnormalities [28]. Moreover, compared with non-ICM, the critical components of VT circuits in ICM are generally located in the sub-endocardial region, resulting in higher ablation success rates [29, 30]. However, patients with ICM often present with extensive myocardial scarring, which may increase the difficulty of intraoperative target mapping [31]. Furthermore, perioperative complications like pericardial effusion and bleeding also need close monitoring [15]. At present, the European Society of Cardiology (ESC) guidelines recommend AADs (Level B) or catheter ablation (Level C) as first-line treatments (a class IIa) for VT in ICM [26]. However, the robust evidence supporting the optimal strategy remains lacking.

Khan *et al*. [32] conducted a meta-analysis comparing catheter ablation with medical therapy for VT in patients with ICM. This analysis found fewer VT storms and ICD shocks in the ablation group, but no significant differences in VT recurrence or all-cause mortality. Our study aligns with theirs on VT recurrence and all-cause mortality, but no statistical differences were observed in VT storms and ICD shocks. Furthermore, our meta-analysis demonstrated that catheter ablation reduced the composite endpoint and adverse events. Notably, our study specifically focused on catheter ablation for documented VT in ICM, excluding primary prophylactic ablation.

Successful ablation sites for VT are predominantly located within the scar border zone defined by substrate voltage mapping [33]. Catheter ablation achieves long-term arrhythmia control by potentially eliminating the arrhythmogenic substrate [34]. In contrast, AADs suppress VT episodes by altering myocardial electrophysiological properties without modifying the underlying pathological substrate of scar-related reentry [35]. Our meta-analysis demonstrates that the ablation group had significantly lower rates of the composite endpoint and cardiovascular hospitalizations than the control group, indicating that targeting the arrhythmogenic substrate can improve clinical outcomes. However, no statistically significant difference was observed in all-cause mortality between the two groups. The possible reason could be that the enrolled patients received an ICD implantation, which may obscure the ablation’s survival benefits by preventing sudden cardiac death [36]. Furthermore, no significant differences were found in VT recurrence or appropriate ICD shocks between groups, which may be attributable to the limited number of studies and their small sample sizes. Trial sequential analysis indicated that future studies should expand sample sizes to investigate VT recurrence and appropriate ICD shocks. Additionally, the timing of endpoint assessment varied across studies: the VANISH series used data collected ≥14 days post-procedure, whereas other trials included earlier postoperative assessments. This early phase is marked by peak inflammatory response and tissue edema in the ablation zone, which can promote electrical instability and increase the incidence of ICD shocks and VT recurrence [37]. Therefore, employing endpoint assessments from ≥14 days after the procedure may provide a more objective evaluation of the long-term efficacy of catheter ablation.

Our study also demonstrated a potential advantage of catheter ablation in reducing adverse events, particularly within the first two years. Catheter ablation avoids drug-related systemic toxicity [28]. Meanwhile, among the six studies, four utilized 3D electroanatomic mapping, which enhances targeting precision, thereby improving procedural success and reducing acute complications [38]. However, we observed significant heterogeneity in adverse events, primarily originating from two studies: In the SMS study [21], ablation was often performed before ICD implantation, making it difficult to distinguish between the independent benefit of the ablation procedure itself and the avoidance of complications related to the ICD device. Additionally, the lack of a uniform ablation technique across the studies may have diminished the apparent treatment effect on adverse events. In the VANISH-2 study [11], adverse events were broadly defined to include both procedure-related events and drug-related toxicities. Furthermore, differences in follow-up duration may be another source of heterogeneity: The risk in the ablation group was concentrated in the short term after the procedure, whereas adverse events in the medication group could accumulate over time. Therefore, future randomized controlled trials with longer follow-up periods, standardized ablation techniques, and well-defined endpoints are needed to evaluate differences in adverse events between the two strategies.

Owing to the complexity of myocardial fibrosis and scar substrate, the management of VT in patients with ICM presents significant challenges [5, 39]. Our meta-analysis reveals the potential of catheter ablation as a first-line therapy, particularly with advances in high-resolution mapping and imaging technologies [40]. Future research should prioritize four domains: Firstly, expanding enrollment to include patients with LVEF ≥35% (as our study was limited to those with LVEF <35%) to define the cardiac function cutoff for ablation benefit, complemented by analyses stratified by scar burden and VT phenotype to guide personalized therapy. Secondly, investigating the effect of catheter ablation on clinical endpoints such as all-cause mortality in patients without ICD implantation. Thirdly, comparing different catheter ablation methods, particularly evaluating the efficacy and safety of the emerging pulsed-field ablation (PFA) for treating VT in patients with ICM. PFA has garnered significant interest due to its relative myocardial specificity and absence of thermal injury [41]; however, its efficacy within scarred myocardium requires further validation. Finally, determining the optimal timing for intervention by directly comparing the long-term survival benefits of prophylactic versus therapeutic ablation strategies

## 5. Limitations

This meta-analysis has some key limitations. First, the limited number of studies and their small sample sizes may increase the risk of selection bias. To address this, future studies with larger sample sizes are required to robustly evaluate the efficacy of catheter ablation. Secondly, there are certain differences in the definitions of the composite endpoint and the adverse events among various studies, which may increase the heterogeneity of the outcomes. Thirdly, the included articles did not limit the classification of VT, the types of pharmacological interventions, and the differences in ablation techniques, which may influence the final outcomes. Fourthly, the heterogeneity of patients, especially the differences in cardiac function status and scar burden, presents challenges in measuring the effectiveness of catheter ablation. Finally, long-term follow-up data are lacking in some studies, which renders it challenging to comprehensively assess the long-term efficacy of catheter ablation.

## 6. Conclusions

This meta-analysis demonstrated that catheter ablation reduced the incidence of the composite endpoint, cardiac hospitalizations, and adverse events for VT in patients with ICM, but there were no statistically significant differences in VT recurrence, appropriate ICD shocks, and all-cause mortality.

## Availability of Data and Materials

All data utilized in this systematic review and meta-analysis were derived from publicly accessible randomized controlled trials (RCTs). The original data of the included RCTs are retrievable through their respective published articles and supplementary materials. All data generated or analyzed as part of the present study have been incorporated into this published article and its accompanying Supplementary Information files.

## Supplementary Material

Supplementary material associated with this article can be found, in the online version, at https://doi.org/10.31083/RCM46164.
