# Supplementary material for: Catheter Ablation vs. Medical Therapy for Ventricular Tachycardia in Ischemic Cardiomyopathy: A Meta-Analysis and Trial Sequential Analysis of Randomized Controlled Trials
Source: Rev Cardiovasc Med. 2026 Feb 26;27(2):46164. doi: 10.31083/RCM46164 (PMC12960011; doi:10.31083/RCM46164)
Supplement: Supplementary file 1 [file 2153-8174-27-2-46164-s1.zip › Supplementary Material.docx]

**Supplementary materials**

**Supplementary Table 1. Search strategy of PubMed database.**

| **#** | **Searches strategy of PubMed** | **Results** |
| --- | --- | --- |
| #1 | myocardial ischemia[MeSH Terms] | 488137 |
| #2 | ((((((((Myocardial Ischemia[Title/Abstract]) OR (Disease, Ischemic Heart[Title/Abstract])) OR (Diseases, Ischemic Heart[Title/Abstract])) OR (Heart Diseases, Ischemic[Title/Abstract])) OR (Ischemic Heart Diseases[Title/Abstract])) OR (Ischemia, Myocardial[Title/Abstract])) OR (Ischemias, Myocardial[Title/Abstract])) OR (Myocardial Ischemias[Title/Abstract])) OR (Ischemic Heart Disease[Title/Abstract]) | 64059 |
| #3 | #1 OR #2 | 510598 |
| #4 | Tachycardia, Ventricular[MeSH Terms] | 20269 |
| #5 | ((((((((((((((Ventricular Tachycardias[Title/Abstract]) OR (Ventricular Tachycardia[Title/Abstract])) OR (Paroxysmal Supraventricular Tachycardia[Title/Abstract])) OR (Paroxysmal Supraventricular Tachycardias[Title/Abstract])) OR (Supraventricular Tachycardia, Paroxysmal[Title/Abstract])) OR (Tachycardia, Paroxysmal Supraventricular[Title/Abstract])) OR (Idiopathic Ventricular Tachycardia[Title/Abstract])) OR (Idiopathic Ventricular Tachycardias[Title/Abstract])) OR (Ventricular Tachycardia, Idiopathic[Title/Abstract])) OR (Nonsustained Ventricular Tachycardia[Title/Abstract])) OR (Nonsustained Ventricular Tachycardias[Title/Abstract])) OR (Tachycardia, Nonsustained Ventricular[Title/Abstract])) OR (Ventricular Tachycardia, Nonsustained[Title/Abstract])) OR (Ventricular Tachyarrhythmias[Title/Abstract])) OR (Tachyarrhythmia, Ventricular[Title/Abstract]) | 33458 |
| #6 | #4 OR #5 | 41004 |
| #7 | catheter ablation[MeSH Terms] | 41946 |
| #8 | (((((((((((((((((((((Ablation, Catheter[Title/Abstract]) OR (Ablation, Transvenous Electric[Title/Abstract])) OR (Electric Ablation, Transvenous[Title/Abstract])) OR (Transvenous Electric Ablation[Title/Abstract])) OR (Ablation, Transvenous Electrical[Title/Abstract])) OR (Electrical Ablation, Transvenous[Title/Abstract])) OR (Transvenous Electrical Ablation[Title/Abstract])) OR (Catheter Ablation, Electric[Title/Abstract])) OR (Electric Catheter Ablation[Title/Abstract])) OR (Ablation, Electric Catheter[Title/Abstract])) OR (Catheter Ablation, Electrical[Title/Abstract])) OR (Ablation, Electrical Catheter[Title/Abstract])) OR (Electrical Catheter Ablation[Title/Abstract])) OR (Catheter Ablation, Percutaneous[Title/Abstract])) OR (Percutaneous Catheter Ablation[Title/Abstract])) OR (Ablation, Percutaneous Catheter[Title/Abstract])) OR (Catheter Ablation, Radiofrequency[Title/Abstract])) OR (Radiofrequency Catheter Ablation[Title/Abstract])) OR (Ablation, Radiofrequency Catheter[Title/Abstract])) OR (Catheter Ablation, Transvenous[Title/Abstract])) OR (Transvenous Catheter Ablation[Title/Abstract])) OR (Ablation, Transvenous Catheter[Title/Abstract]) | 5784 |
| #9 | #7 OR #8 | 43414 |
| #10 | ((randomized controlled trial[Title/Abstract]) OR (randomized[Title/Abstract])) OR (placebo[Title/Abstract]) | 880674 |
| #11 | #3 AND #6 AND #9 AND #10 | 40 |

**Supplementary Table 2. Search strategy of EMBASE database.**

| **#** | **Searches strategy of EMBASE** | **Results** |
| --- | --- | --- |
| #1 | 'ischemic heart disease'/exp | 843189 |
| #2 | 'coronary artery insufficiency':ab,ti OR 'coronary heart disease':ab,ti OR 'coronary insufficiency':ab,ti OR 'heart disease, coronary':ab,ti OR 'heart disease, ischaemic':ab,ti OR 'heart disease, ischemic':ab,ti OR 'ischaemia heart disease':ab,ti OR 'ischaemic cardiac disease':ab,ti OR 'ischaemic cardial disease':ab,ti OR 'ischaemic cardiopathy':ab,ti OR 'ischaemic heart disease':ab,ti OR 'ischemia heart disease':ab,ti OR 'ischemic cardiac disease':ab,ti OR 'ischemic cardial disease':ab,ti OR 'ischemic cardiopathy':ab,ti OR 'ischemic heart disease':ab,ti | 143516 |
| #3 | #1 OR #2 | 880269 |
| #4 | 'heart ventricle tachycardia'/exp | 57333 |
| #5 | 'ventricular tachycardias':ab,ti OR 'ventricular tachycardia':ab,ti OR 'paroxysmal supraventricular tachycardia':ab,ti OR 'paroxysmal supraventricular tachycardias':ab,ti OR 'supraventricular tachycardia, paroxysmal':ab,ti OR 'tachycardia, paroxysmal supraventricular':ab,ti OR 'idiopathic ventricular tachycardia':ab,ti OR 'idiopathic ventricular tachycardias':ab,ti OR 'ventricular tachycardia, idiopathic':ab,ti OR 'nonsustained ventricular tachycardia':ab,ti OR 'nonsustained ventricular tachycardias':ab,ti OR 'tachycardia, nonsustained ventricular':ab,ti OR 'ventricular tachycardia, nonsustained':ab,ti OR 'ventricular tachyarrhythmias':ab,ti OR 'tachyarrhythmia, ventricular':ab,ti | 49710 |
| #6 | #4 OR #5 | 72635 |
| #7 | 'catheter ablation'/exp | 47484 |
| #8 | 'ablation, catheter':ab,ti OR 'catheter ablation, electric':ab,ti OR 'electric catheter ablation':ab,ti OR 'ablation, electric catheter':ab,ti OR 'catheter ablation, electrical':ab,ti OR 'ablation, electrical catheter':ab,ti OR 'electrical catheter ablation':ab,ti OR 'catheter ablation, percutaneous':ab,ti OR 'percutaneous catheter ablation':ab,ti OR 'ablation, percutaneous catheter':ab,ti OR 'catheter ablation, radiofrequency':ab,ti OR 'radiofrequency catheter ablation':ab,ti OR 'ablation, radiofrequency catheter':ab,ti OR 'catheter ablation, transvenous':ab,ti OR 'transvenous catheter ablation':ab,ti OR 'ablation, transvenous catheter':ab,ti OR 'ablation, transvenous electric':ab,ti OR 'electric ablation, transvenous':ab,ti OR 'transvenous electric ablation':ab,ti OR 'ablation, transvenous electrical':ab,ti OR 'electrical ablation, transvenous':ab,ti OR 'transvenous electrical ablation':ab,ti OR 'catheter ablation':ab,ti | 31084 |
| #9 | #7 OR #8 | 52002 |
| #10 | ((randomized controlled trial[Title/Abstract]) OR (randomized[Title/Abstract])) OR (placebo[Title/Abstract]) | 1253562 |
| #11 | #3 AND #6 AND #9 AND #10 | 105 |

**Supplementary Table 3. Search strategy of Cochrane Library database.**

| **#** | **Searches strategy of Cochrane Library** | **Results** |
| --- | --- | --- |
| #1 | MeSH descriptor: [Myocardial Ischemia] explode all trees | 38481 |
| #2 | (Myocardial Ischemia):ti,ab,kw OR (Disease, Ischemic Heart):ti,ab,kw OR (Diseases, Ischemic Heart):ti,ab,kw OR (Heart Diseases, Ischemic):ti,ab,kw OR (Ischemic Heart Diseases):ti,ab,kw OR (Ischemia, Myocardial):ti,ab,kw OR (Ischemias, Myocardial):ti,ab,kw OR (Myocardial Ischemias):ti,ab,kw OR (Ischemic Heart Disease):ti,ab,kw | 21736 |
| #3 | #1 OR #2 | 50171 |
| #4 | MeSH descriptor: [Tachycardia, Ventricular] explode all trees | 991 |
| #5 | (Ventricular Tachycardias):ti,ab,kw OR (Ventricular Tachycardia):ti,ab,kw OR (Paroxysmal Supraventricular Tachycardia):ti,ab,kw OR (Paroxysmal Supraventricular Tachycardias):ti,ab,kw OR (Supraventricular Tachycardia, Paroxysmal):ti,ab,kw OR (Tachycardia, Paroxysmal Supraventricular):ti,ab,kw OR (Idiopathic Ventricular Tachycardia):ti,ab,kw OR (Idiopathic Ventricular Tachycardias):ti,ab,kw OR (Ventricular Tachycardia, Idiopathic):ti,ab,kw OR (Nonsustained Ventricular Tachycardia):ti,ab,kw OR (Nonsustained Ventricular Tachycardias):ti,ab,kw OR (Tachycardia, Nonsustained Ventricular):ti,ab,kw OR (Ventricular Tachycardia, Nonsustained):ti,ab,kw OR (Ventricular Tachyarrhythmias):ti,ab,kw OR (Tachyarrhythmia, Ventricular):ti,ab,kw | 3696 |
| #6 | #4 OR #5 | 3734 |
| #7 | MeSH descriptor: [Catheter Ablation] explode all trees | 2330 |
| #8 | (Ablation, Catheter):ti,ab,kw OR (Ablation, Transvenous Electric):ti,ab,kw OR (Electric Ablation, Transvenous):ti,ab,kw OR (Transvenous Electric Ablation):ti,ab,kw OR (Ablation, Transvenous Electrical):ti,ab,kw OR (Electrical Ablation, Transvenous):ti,ab,kw OR (Transvenous Electrical Ablation):ti,ab,kw OR (Catheter Ablation, Electric):ti,ab,kw OR (Electric Catheter Ablation):ti,ab,kw OR (Ablation, Electric Catheter):ti,ab,kw OR (Catheter Ablation, Electrical):ti,ab,kw OR (Ablation, Electrical Catheter):ti,ab,kw OR (Electrical Catheter Ablation):ti,ab,kw OR (Catheter Ablation, Percutaneous):ti,ab,kw OR (Percutaneous Catheter Ablation):ti,ab,kw OR (Ablation, Percutaneous Catheter):ti,ab,kw OR (Catheter Ablation, Radiofrequency):ti,ab,kw OR (Radiofrequency Catheter Ablation):ti,ab,kw OR (Ablation, Radiofrequency Catheter):ti,ab,kw OR (Catheter Ablation, Transvenous):ti,ab,kw OR (Transvenous Catheter Ablation):ti,ab,kw OR (Ablation, Transvenous Catheter):ti,ab,kw | 4494 |
| #9 | #7 OR #8 | 4505 |
| #10 | #3 AND #6 AND #9 | 76 |

**Supplementary Table 4.** the definition of composite endpoint

| VANISH  2016 | The primary outcome was all-cause mortality at any time or ventricular tachycardia storm or appropriate shock from an ICD after the 30-day treatment period. |
| --- | --- |
| SURVIVE-VT  2022 | The primary outcome was cardiovascular death, appropriate ICD shock, unplanned hospitalization for worsening heart failure or severe treatment-related complications from enrollment up to the 24-month follow-up |
| VANISH-2  2024 | The primary endpoint was a composite of all-cause mortality from any cause during follow-up or, more than 14 days after randomization, appropriate ICD shock, ventricular tachycardia storm (at least three ventricular tachycardia events within 24 hours), or treated sustained ventricular tachycardia below the detection limit of the ICD |

**Supplementary Table 5.** Subgroup analysis of adverse events

| **Subgroup** | **Grouping Status** | **Number of studies** | **Meta-analysis** | | | **Heterogeneity** | | **P-value of**  **difference between subgroups** |
| --- | --- | --- | --- | --- | --- | --- | --- | --- |
|  |  |  | **Effect size** | **95%CI** | **P-value** | **I^2^** | **P-value** |  |
| LVEF | ＜25% | 1 | 0.65 | 0.19-2.18 | P=0.48 | - | - | 0.81 |
|  | ＞25% | 5 | 0.75 | 0.62-0.91 | P=0.004 | 73% | P=0.005 |  |
| drug interventions | AADs | 4 | 0.69 | 0.54-0.87 | P=0.002 | 65% | P=0.04 | 0.09 |
|  | Non-AADs | 2 | 0.96 | 0.71-1.29 | P=0.77 | 56% | P=0.13 |  |
| Follow-up time | >2years | 2 | 0.98 | 0.78-1.22 | P=0.83 | 0% | P=0.42 | 0.0005 |
|  | <2years | 4 | 0.46 | 0.32-0.66 | P＜0.001 | 0% | P=0.82 |  |

**Supplementary Table 6.** other outcomes

| **outcome** | **Number of studies** | **Simple size** | **Meta-analysis** | | | **Heterogeneity** | |
| --- | --- | --- | --- | --- | --- | --- | --- |
|  |  |  | **Effect size** | **95%CI** | **P-value** | **I^2^** | **P-value** |
| VT storms | 5 | 1037 | 0.81 | 0.65-1.00 | P=0.89 | 0% | P=0.05 |
| Inappropriate ICD shocks | 3 | 510 | 0.90 | 0.50-1.63 | P=0.62 | 0% | P=0.73 |
| Cardiac death | 6 | 1064 | 1.11 | 0.83-1.49 | P=0.87 | 0% | P=0.46 |
| Syncope | 3 | 634 | 0.78 | 0.40-1.52 | P=0.23 | 31% | P=0.64 |

**Supplementary Table 7.** GRADE evidence


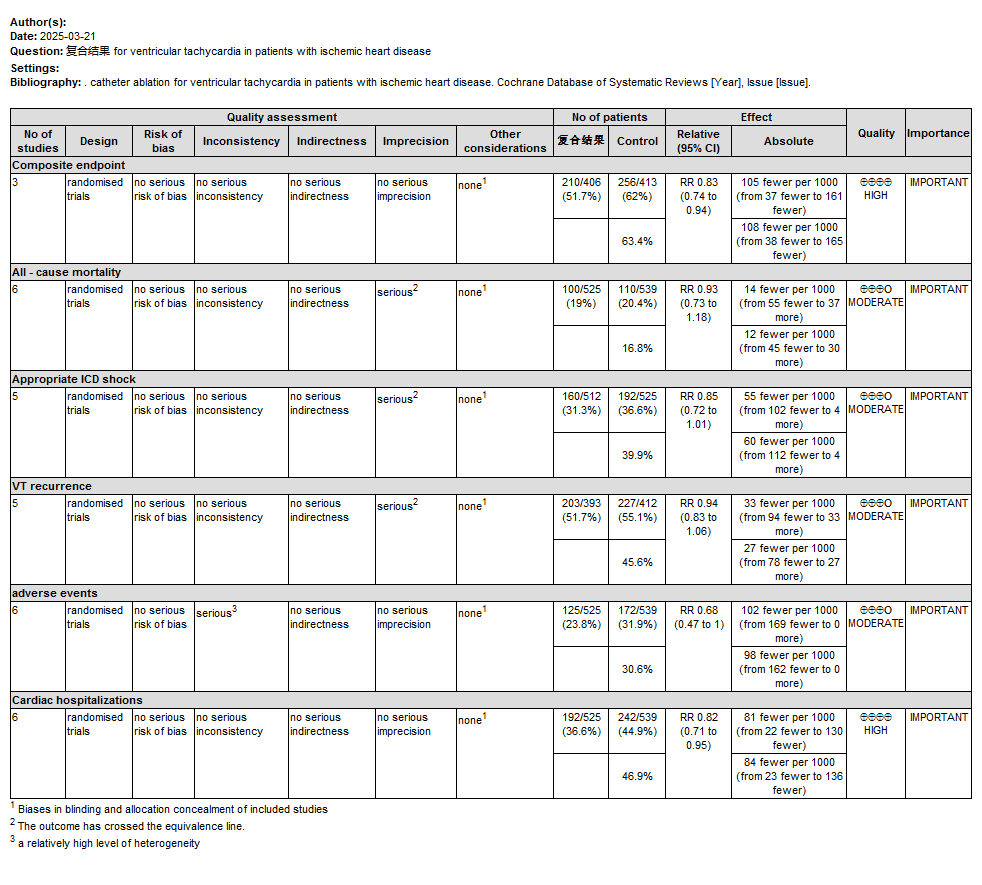


**Supplementary Fig. 1.** The quality assessment of RCT


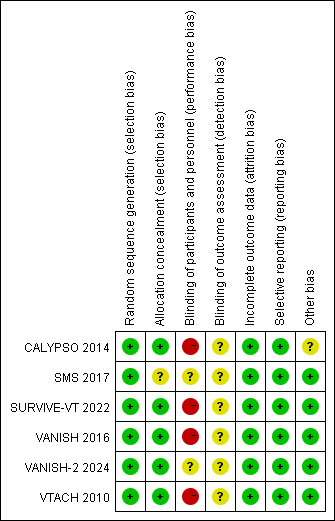


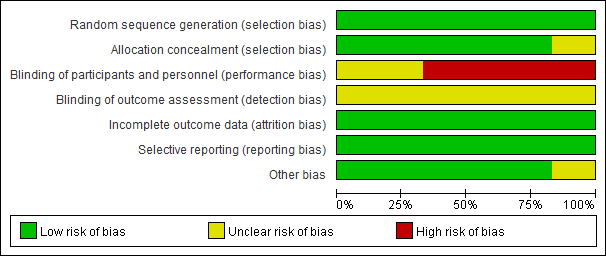


**Supplementary Fig. 2.** Trial sequential analysis (TSA)


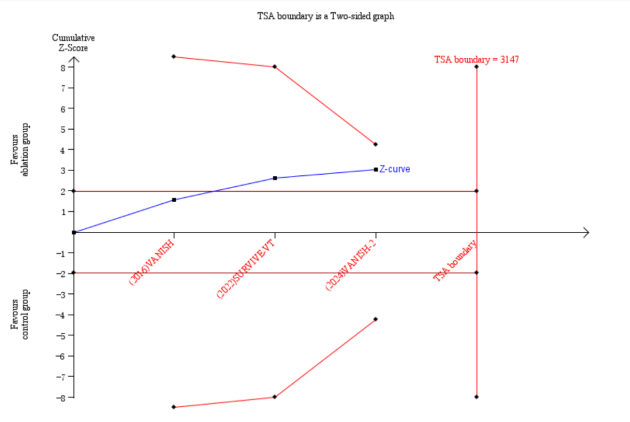

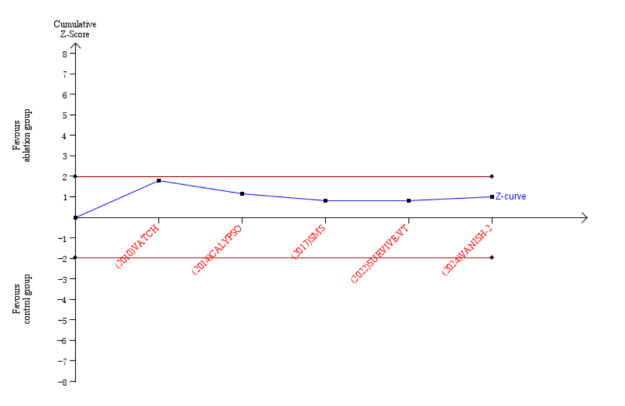


a.composite endpoint b.VT recurrence


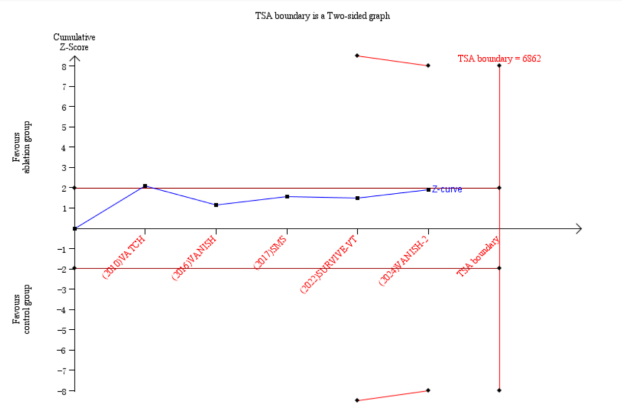

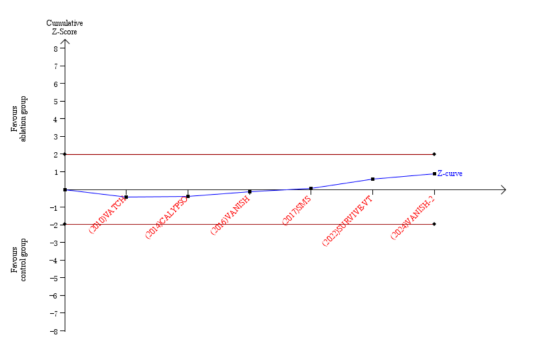


c.appropriate ICD shocks d.All - cause mortality


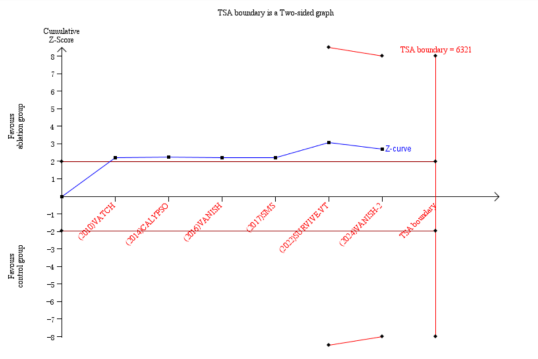

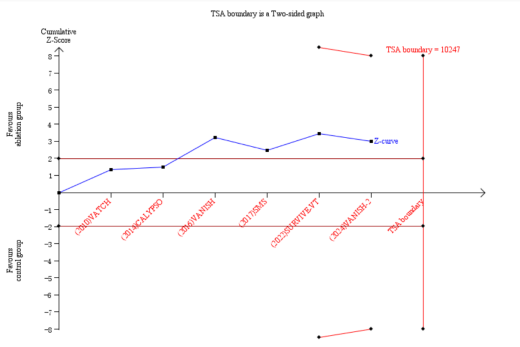


e.cardiac hospitalizations f.adverse events

**Supplementary Fig. 3.** funnel plots.


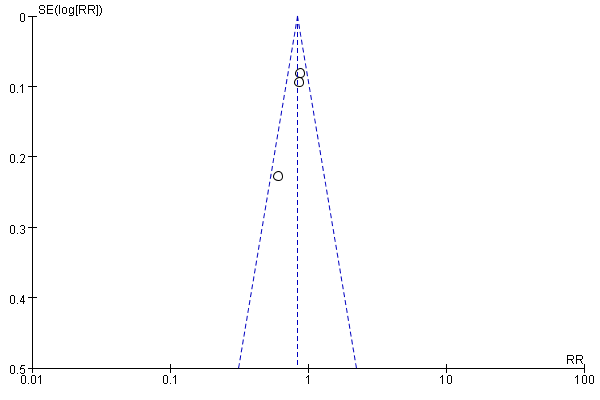

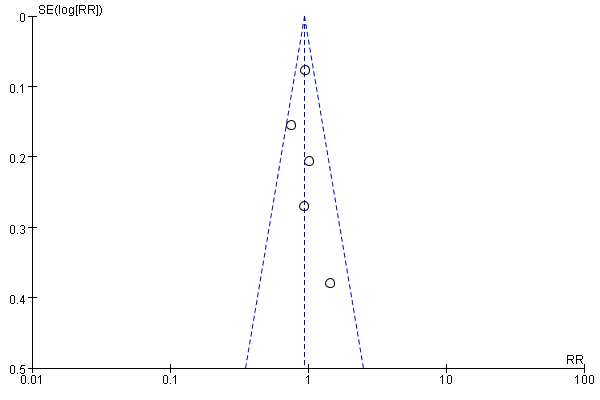


a.composite endpoint b.VT recurrence


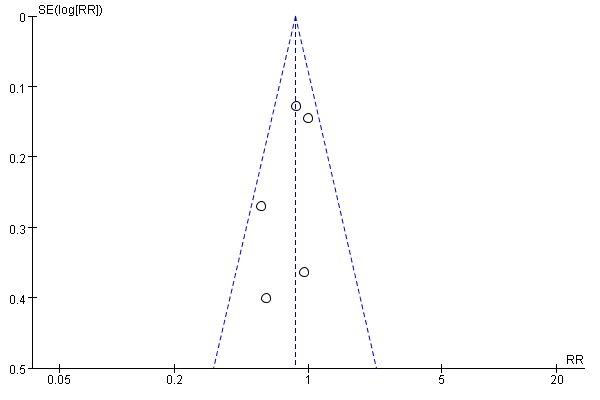

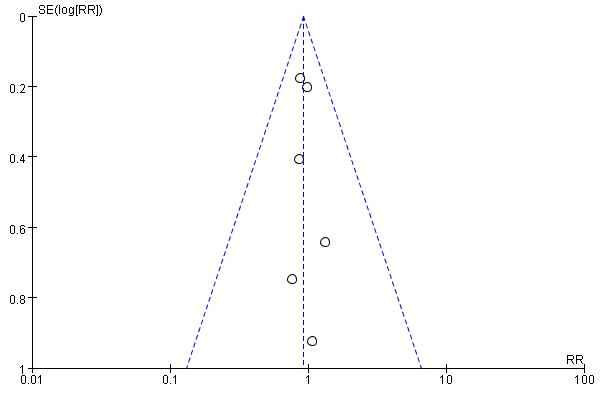


c.appropriate ICD shocks d.All - cause mortality


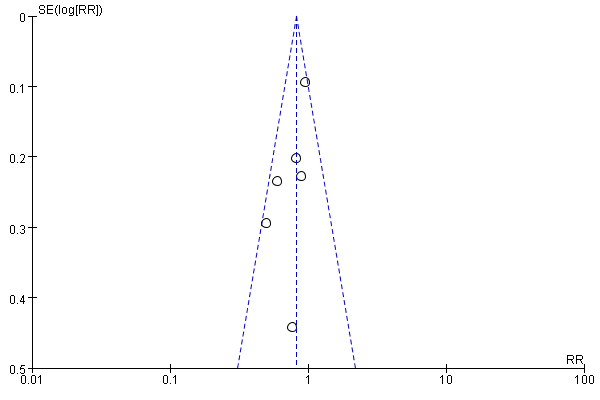

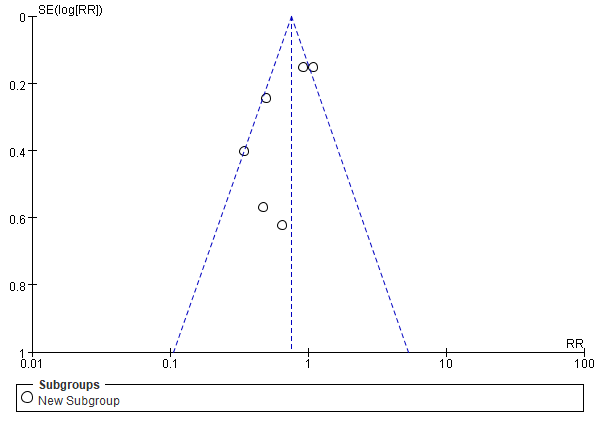


e.cardiac hospitalizations f.adverse events
